# I2 Statistic as the Selection Bias Test: Trial Effect Estimates in Relation to Identified Bias Levels

**DOI:** 10.7759/cureus.94704

**Published:** 2025-10-16

**Authors:** Steffen Mickenautsch, Veerasamy Yengopal

**Affiliations:** 1 Faculty of Dentistry, University of the Western Cape, Cape Town, ZAF; 2 Community Dentistry, University of the Witwatersrand, Johannesburg, ZAF

**Keywords:** bias test, clinical trial appraisal, randomised control trials, selection bias, systematic review and meta-analysis

## Abstract

Aim: This study aimed to investigate the association between selection bias, established by the use of the I^2^ test from published randomised controlled trials (RCTs), and the effect estimate magnitude of these trials. Two null hypotheses were tested: H01: The magnitude of trial effect estimates is not significantly positively correlated with the identified selection bias levels. H02: The magnitude of trial effect estimates does not differ significantly between RCTs with identified ‘low’ and ‘high’ selection biases.

Methods: RCTs reporting computable outcomes and baseline data were selected from published systematic review reports that, in turn, were identified through a systematic literature search in PubMed up to 2024. All RCTs were tested for selection bias using the trial-adjusted, simulated comparator trial (SCT)-based I^2^ test. For each RCT, the selection bias level (B%) was determined, and the absolute value of the risk difference (RD) point estimate was calculated. H01 was tested using Spearman’s rank correlation, and H02 was tested using an independent samples t-test. A sensitivity analysis was carried out to examine any potential confounder effect.

Results: A total of 332 RCTs, published from 1985 to 2023 in various medical specialties, were tested. Test applicability was limited by the low quality of RCT reporting. ‘Low’ selection bias was identified in 202 RCTs and ‘high’ selection bias in 130 RCTs. The estimation of selection bias levels within pre-specified I^2^ point estimate thresholds was possible for 71% of all RCTs. For 97 (29%) RCTs, the computed I^2^ point estimates fell outside these thresholds, and therefore, B% estimation was possible by approximation only. Such an approximation proved to have a confounding effect. After confounder correction, there was a significant positive correlation between the magnitudes of trial effect estimates and the selection bias levels (Spearman’s r = 0.25, p < 0.001). The effect estimates were statistically significantly higher (0.07; 95%CI: 0.03-0.11; p = 0.0005) for RCTs with identified ‘high’ selection bias than for RCTs with ‘low’ selection bias, representing a proportional over-estimation of 64%. Both null hypotheses H01 and H02 were rejected.

Conclusion: The trial-adjusted, SCT-based I^2^ test appeared to be effective for identifying high-level selection bias in RCTs. The test may allow estimation of the selection bias extent and its possible effect on the reported trial effect estimate.

## Introduction

Mickenautsch and Yengopal (2024) adopted the I^2^ statistic [1] for use as a selection bias test in single randomised controlled trials (RCTs). Heterogeneity in baseline variables, included in a meta-analysis, should always be zero because they do not share population or intervention differences. Hence, the only plausible explanation for heterogeneity in baseline variables is poor randomisation. When the I^2^ point estimate is used in a baseline variable meta-analysis as a measure, baseline imbalances caused by non-random allocation of patients to intervention groups will deviate from a zero I^2^ value and thus indicate the presence of selection bias [2]. In order to apply the test, data from one baseline variable, selected for being predictive of the trial’s measured outcome and reported in the RCT, are extracted and utilised to generate two ‘simulated comparator trials’ (SCTs) that are sufficiently similar to the RCT. The generated data from both SCTs, together with that of the RCT’s baseline variable, consisting of mean value with standard deviation (SD) and sample size for both test and control groups, are pooled in a fixed-effect baseline variable meta-analysis, and the resulting I^2^ point estimate is noted. A point estimate of I^2^ = 0% indicates the absence, and any point estimate of I^2^ > 0%, the presence of selection bias in the RCT [3,4].

Thus far, the current version of the test [2] did not specify the extent of the identified selection bias for an RCT nor the possible effect that such bias on the reported trial effect estimate may have had. Therefore, it was impossible to determine how much the bias had diverted the reported trial effect estimate from the true treatment effect.

In a subsequent investigation, a varying relationship between the I^2^ point estimate and trial sample size, unique for each of 11 different, simulated selection bias levels (B% = 0, 10, 20, 30, 40, 50, 60, 70, 80, 90, and 100), was noted. Under the RCT simulation, these bias levels represent the estimated percentage (B%) of trial subjects from the total RCT sample size (n_i_) that were non-randomly allocated in favour of one treatment group over the other [5].

In addition, the influence of the sample size (n_i_) beside the influence of the level of selection bias on the I^2^ point estimate was observed [5]. Accordingly, when the I^2^ point estimate was obtained in a baseline variable meta-analysis specifically for n_i_ = 10, 50, and 100 per group for the bias levels B% = 40, 50, 60, 70, 80, 90 and 100, as well as for bias levels B% = 0, 10, 20, and 30 with artificially highly increased sample sizes n_i_ = 5,000, 18,000, and 36,000, I^2^ point estimate values were identified that were specific for each of the 11 bias levels. For example, if the pooling of baseline variable values yielded an I^2^ point estimate of 0%, 40%, and 72% for sample sizes 10, 50, and 100, respectively, then these corresponded with bias level B% = 50, indicating that between 41% and 50% of all trial subjects were non-randomly allocated in favor of one treatment group above the other [5].

Furthermore, a simulation study found that the percentage of trials with statistically significant effect estimates, due to selection bias alone, increased from zero at bias levels B% = 0, 10, 20 or 30 to 100% at B% = 40, 50, 60, 70, 80, 90 or 100 [6]. Based on this observed difference, the practical distinction between a ‘low’ (B% = 0- 30) versus ‘high’ (B% = 40-100) selection bias effect, based on I^2^ test results, may be justified.

These trial simulation results [5,6] raise the question of whether a B%-dependent bias effect on the effect estimates of published RCTs may also be observed. For this reason, the aim of our study was to investigate the association between the extent of selection bias in published RCTs and the magnitude of their reported effect estimates. The objectives were to test the two null-hypotheses that the magnitude of trial effect estimates is not significantly positively correlated with the identified selection bias level spectrum (B% = 0-100) (H01) and that the magnitude of trial effect estimates does not differ significantly between RCTs with identified ‘low’ (B% 0-30) and ‘high’ (B% 40-100) selection biases (H02).

This manuscript has been published as a preprint on Authorea on September 17, 2025 (DOI: 10.22541/au.175812559.92131164/v1) [7].

## Materials and methods

Suitable RCTs were selected from published systematic review reports that were identified through a systematic literature search. Although this investigation followed a meta-epidemiological study design, adjusted for null-hypothesis testing and not a systematic review, it was reported, as much as possible, in line with the Preferred Reporting Items for Systematic Reviews and Meta-Analyses (PRISMA) statement (Appendices - Section 1) [8]. During the study, several changes were made to the applied study methodology from the original published study protocol [9]: a higher number of systematic review reports were provisionally included, and a sensitivity analysis of the main study results was added (Appendices - Section 2).

Selection of systematic review reports

All 467 citations of systematic review reports identified through a systematic literature search in a previous study were provisionally included for full-copy article tracing [10]. These citations were identified through a PubMed search until January 24, 2024, using the search string: (systematic review rob 2 OR Cochrane RoB 2.0 OR Cochrane RoB 2) AND systematic review with the following set limits: Article type = Systematic review; Publication date = 1 year. The full references of all reports, together with their identification numbers, were listed in a separate MS Excel data sheet (Microsoft Corp., USA).

All full copies of the traced systematic review reports were screened. Reports were excluded when there was no clear reporting or lack of reporting regarding version 2 of Cochrane’s Risk of Bias (RoB 2) tool/domain 1 assessment [11], concerning selection bias risk; no reporting of overall RoB 2 ratings; and when version 1 instead of version 2 of Cochrane’s RoB tool was used in the review.

In accordance with the sample size calculation results regarding the minimum required number of RCTs for this study, a suitable number of systematic review reports were randomly selected from the total number of the provisionally included full reports. The details of the random selection method are presented in Appendices - Section 3.

All randomly selected systematic review reports were further reviewed. Reports were further excluded according to the following exclusion criteria: duplication, no computable outcome data per RCT reported (outcome data were considered computable when including the number of events (n) and the total number of treated patients (N) per treatment group, and reports without accessible RoB 2 ratings per RCT that were missed during the initial screening process were also excluded.

RCT selection from the included systematic review reports

From the included systematic review reports, all RCTs were selected for which treatment data, including the number of events (n) and the total number of treated patients (N) per treatment group, were reported. No limits concerning the publication language of RCTs were set. The full references of all selected RCTs were recorded from the systematic review reports’ reference lists.

RCT review, outcome, and baseline data extraction and computation

All RCTs identified in the systematic review reports were included for full copy tracing. All traced full RCT copies were reviewed. RCTs were excluded if no full article reference was reported in the systematic review; reported baseline data was not computable (baseline data was only considered computable when reported as mean value with standard deviation (SD) or standard error (SE), or as median value with minimum/maximum range or interquartile range (IQR) and with sample size per group); if no baseline data was reported for at least two randomised treatment groups; when the published RCT report was retracted by the journal; when the group allocation was not clearly reported; and when the sample sizes of the compared treatment groups were not the same as those of the randomised groups at baseline. RCTs that did not follow a parallel group design or cluster RCTs were also excluded.

For each included RCT, all treatment data per measured outcome were extracted from the systematic review report, either from presented forest plots or from the text, including the number of events (n) and sample size (N) for the test and control groups. The baseline data for these groups, consisting of the mean value with the SD or SE or median value with the minimum/maximum range or IQR and sample size, were directly extracted from each RCT report for one selected baseline variable. The baseline variable with the apparent largest difference between the two treatment groups was selected, especially if it was potentially highly predictive of the measured trial outcome(s). All reported SE values were converted to SD using the formula: $SD = SE x \sqrt{N}$ and median values with the minimum/maximum range or IQR were converted to mean (SD) values using the formulas by Hozo et al. (2005) [12] and Wan et al. (2014) [13], respectively.

From the extracted n/N outcome values per treatment group, the risk difference (RD) with 95% confidence interval (CI) was computed. Because the study aimed to assess the selection bias influence on the reported treatment effect regardless of the direction that such an effect may have had, which may alter the RD point estimate between negative and positive values, the RD point estimate’s absolute value was used. If more than one measured outcome was reported per trial, the one with the highest absolute RD point estimate value was selected for data analysis. RD computation from the extracted data was conducted using Cochrane's Review Manager (RevMan) software [14].

One reviewer (SM) selected all systematic reviews and RCT reports and extracted and entered all data into an MS Excel sheet. A second reviewer (VY) reviewed and verified the report selection and data entry for accuracy. Disagreements were resolved via discussion and consensus.

RCT selection bias test

All RCTs underwent selection bias testing using the trial-adjusted, SCT-based I^2^ test, as reported by Mickenautsch and Yengopal, elsewhere [2-5]. A detailed step-by-step description of the test procedure followed is presented in Appendices - Section 1. The extent of selection bias in the tested RCTs was estimated in line with 11 bias levels (B% = 0, 10, 20, 30, 40, 50, 60, 70, 80, 90, 100), reflecting the estimated percentage of subjects non-randomly allocated in favour of one treatment group over the other [5]. The bias levels B% = 0-30 and B% = 40-100 were classified as indicating ‘low’ and ‘high’ selection bias, respectively.

**Figure 1:**
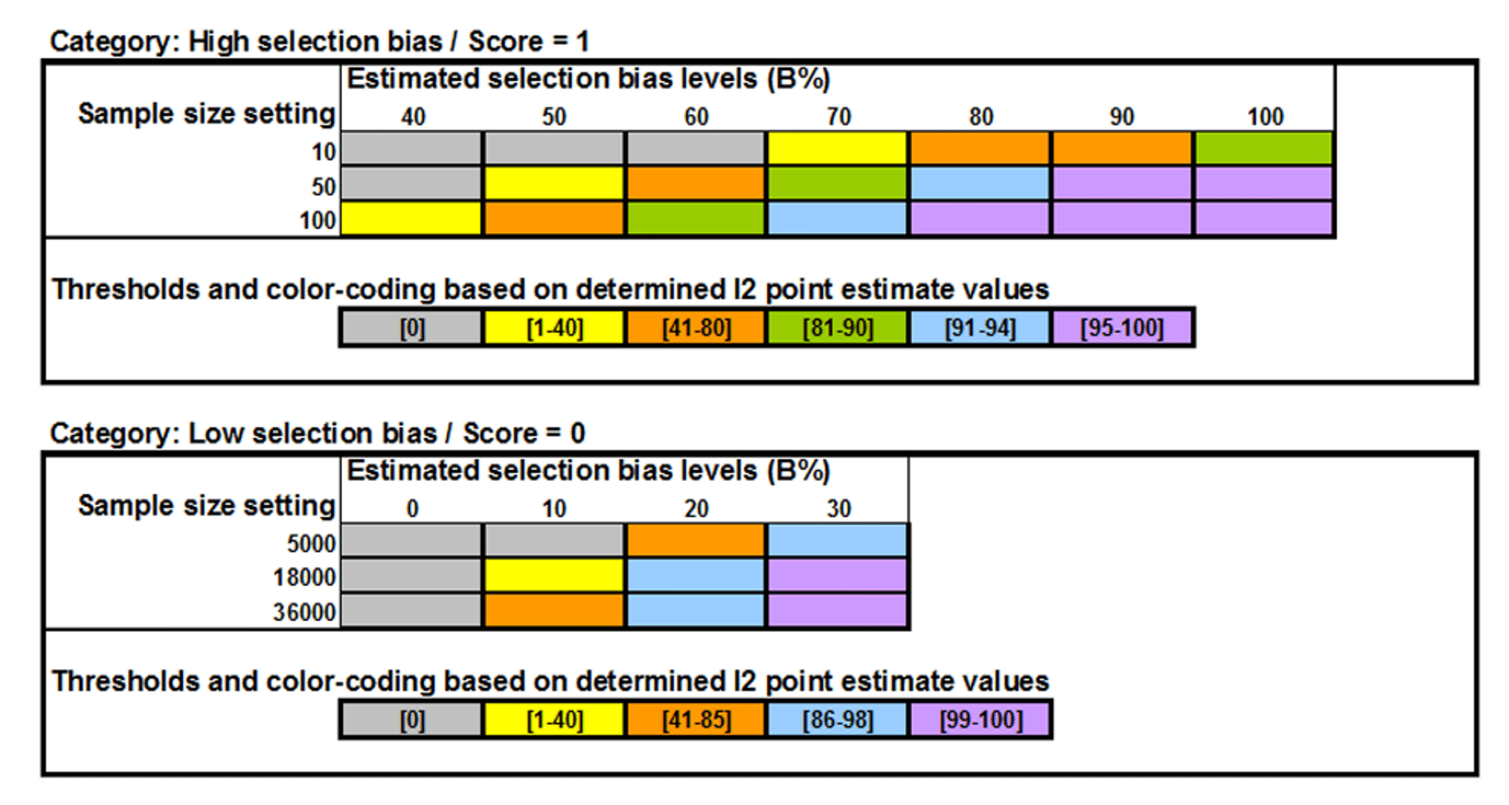
Estimated selection bias levels Image created with MS PowerPoint 2000 SR-1 9.0.3821 (rendered with Adobe Photoshop Elements 11.0)

All bias levels were determined by generating two RCT-adjusted SCTs. The data of both SCTs, consisting of mean (SD) values and sample size (n_i_) for two treatment groups, were entered into a fixed-effect meta-analysis for continuous data and pooled using the RevMan software [14]. This meta-analysis was always expected to generate a zero I^2^ point estimate, which was subsequently confirmed for each tested RCT. The RCT baseline data were added to the analysis, and the n_i_ values were set at 10, 50 and 100 for all treatment groups in both SCTs and the RCT. The meta-analysis was repeated for each of the three sample size settings, and the resulting I^2^ point estimates were recorded for each. In the event that all three settings generated a zero I^2^ point estimate, then all n_i_ values were set at the artificially inflated sample sizes 5,000, 18,000 and 36,000, and the meta-analysis were repeated for each setting. In line with previous findings [5], bias levels were estimated according to pre-specified colour-coded thresholds presented in Figure 1.

Sample size calculation

Sample size calculations for the required minimum number of RCTs to be tested were carried out in G*Power (Heinrich-Heine Universität, Düsseldorf, Germany) [15]. For H01, a medium effect size was assumed (r = 0.5) at a 95% confidence level, thus requiring a minimum sample size of 84 RCTs. For H02 (requiring an independent samples t-test), a medium effect size was also assumed, requiring Cohen’s d = 0.8 at the 95% confidence level and a minimum sample size of 128 RCTs. Due to the lack of prior investigations on this topic and therefore due to the absence of any data to justify the extremes of small or large effect sizes, a medium effect size was chosen as a rational choice for both hypotheses. Hence, a minimum sample size of 128 RCTs for both null hypotheses was accepted for this study.

Main analysis and hypothesis testing

The relationship between the magnitude of the RCT effect estimates (the absolute values of the RD point estimates) and the bias levels (B%) (H01) was determined by Spearman’s rank correlation coefficient. The established bias levels (B%) formed the independent (x) and the absolute values of the RD point estimates from the RCTs, the dependent variable (y), for analysis. The difference between the magnitude of the trial effect estimates (absolute values of RD) for RCTs with defined ‘low’ and ‘high’ selection bias (H02) was determined by the independent samples t-test. The alpha level was set at 5%. The analyses were carried out in SAS (SAS Institute, Cary, North Carolina, USA). 

Sensitivity analysis

In the event that one or both null hypotheses were rejected, sensitivity analysis was conducted to examine the relationship between other potential confounding factors and the effect estimate magnitude, which may explain the changes in the effect estimate independently from that of the selection bias levels (B%). Potential confounding factors were identified graphically using a simple relationship diagram consisting of nodes and edges, where nodes represented various factors related to the effect estimate magnitude, and the edges depicted the relationships between the nodes.

The relationships between the identified factors were tested using Spearman’s correlation and a two-tailed t-test.

## Results

Systematic review report and RCT selection

From the original 467 systematic review citations [10], three could not be traced in full copy and full copies of the remaining 464 systematic review reports were screened. During screening, 41 reports were excluded due to various reasons: no clear reporting of RoB 2/domain 1 assessment (n = 1), no information on bias risk assessment (n = 1), no overall RoB 2 ratings reported (n = 15), not a systematic review (n = 2), RoB 1 used instead of RoB 2 (n = 17), unreadable RoB 2 graph (n = 2), inaccessible supporting material on bias appraisal (n = 2), and unclear reporting of bias appraisal method (n = 1). This led to a provisional inclusion of 423 systematic review reports.

From the 423 provisionally included systematic review reports, 141 reports (33.33%) were randomly selected for further review. Of these, 45 were excluded due to the following reasons: duplication (n = 1), no computable trial outcome data reported (n = 25), no RoB 2 ratings reported per trial (n = 17), and RoB 2 ratings per trial not accessible (n = 2). This resulted in 96 systematic review reports being included for RCT data extraction. The full references of included and excluded reports, along with exclusion reasons, are listed in Appendices - Section 3.

**Figure 2:**
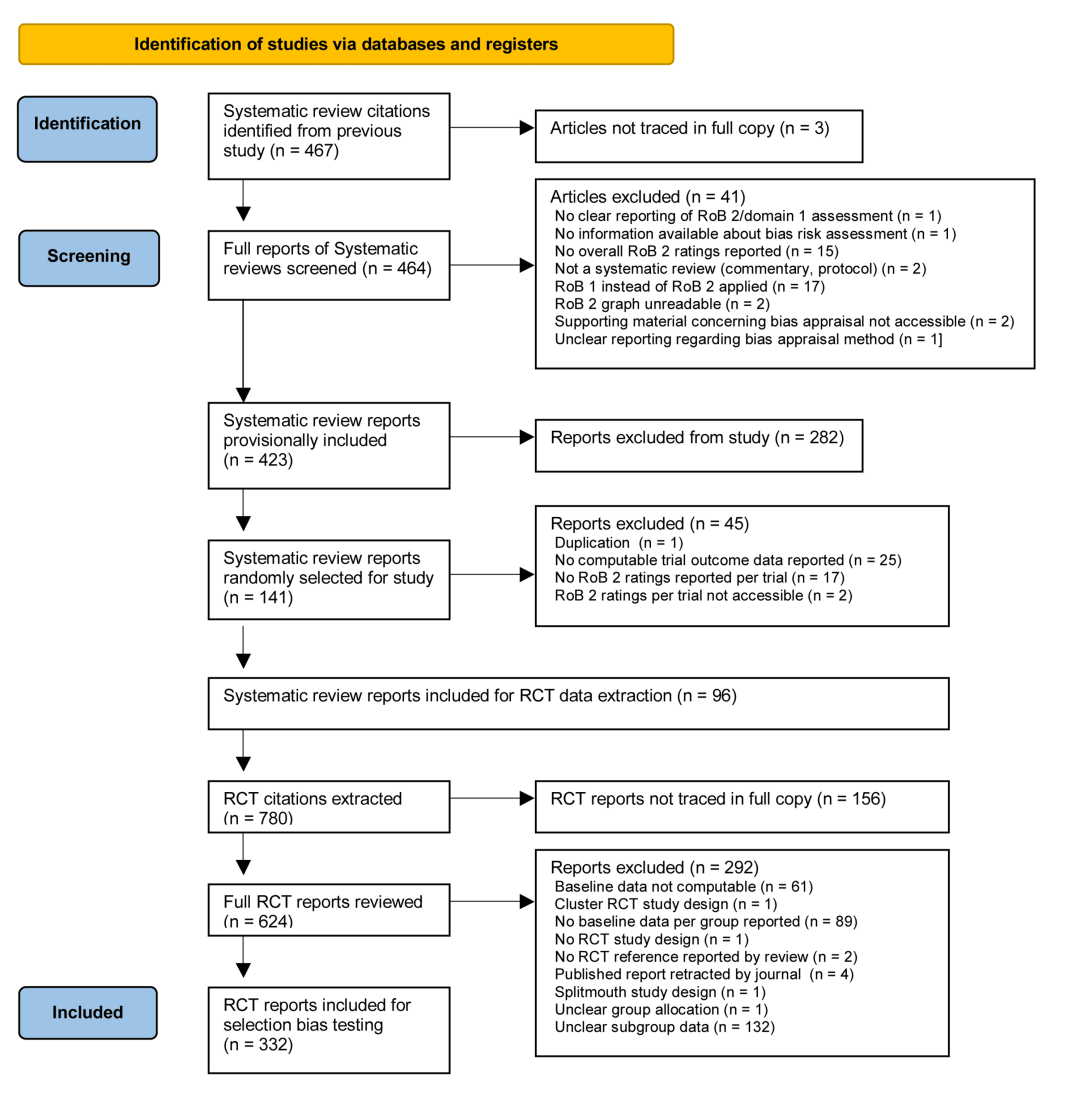
Preferred Reporting Items for Systematic Reviews and Meta-Analyses (PRISMA) flow diagram for systematic review and RCT selection

From the 96 included systematic review reports, a total of 780 RCT citations were extracted. Of these, 156 RCT reports could not be traced in full copy. From the provisionally included 624 RCT reports, 292 were excluded for the following reasons: baseline data not computable (n = 61), cluster RCT study design (n = 1), no baseline data per group reported (n = 89), no RCT study design (n = 1), no RCT reference reported by systematic review (n = 2), published report retracted by journal (n = 4), split-mouth study design (n = 1), unclear group allocation (n = 1), and unclear subgroup data/sample sizes of the compared treatment groups were not the same as that of the randomised groups at baseline (n = 132). A total of 332 RCTs were included for selection bias testing, providing 2.6 times more trials than the minimum required sample size for this study. The full references of all included and excluded RCTs, together with the reasons for exclusion, are listed in Appendices - Section 4. The PRISMA diagram illustrating the systematic review and RCT selection process is presented in Figure 2.

Trial characteristics of the included RCTs

The 332 included RCT reports were published from 1985 to 2023, with a median publication year was 2017, and most reports were published in 2021 (n = 51). The trials were conducted in various medical specialities: internal medicine (n = 112), obstetrics (n = 57), oncology (n = 37), surgery (n = 37), anaesthesiology (n = 30), dentistry (n = 11), psychology (n = 10), physiotherapy (n = 9), preventive medicine (n = 7), nutrition (n = 6), paediatrics (n = 6), ophthalmology (n = 5), neurology (n = 4) and urology (n = 1). According to the systematic review of authors' assessment using the RoB 2 tool, the selection bias risk appraisal of the 332 RCTs indicated that 237 RCTs had 'low bias risk', 80 RCTs had 'some concerns', and 15 RCTs had 'high bias risk' (Appendices - Section 4). 

Main analysis results

The individual results of the selection bias test and absolute RD values for each RCT are presented in Appendices - Section 5. ‘Low’ selection bias was identified in 202 RCTs and ‘high’ selection bias in 130 RCTs. Compared with the selection bias risk assessment by systematic review authors, the risk of not identifying RCTs with elevated selection bias risk (‘high’ or with ‘some concerns’) when using the RoB 2 tool appeared to be 27% higher (relative risk, RR: 0.73; 95% CI: 0.58-0.91; p = 0.005) than when the trial adjusted, where the SCT-based I^2^ test was used.

The estimation of selection bias levels (B%) within the pre-specified I^2^ point estimate thresholds (Figure 1) was possible for 235 (71%) of all RCTs. For 97 (29%) RCTs, the computed I^2^ point estimates fell outside these thresholds for one or two of the three specified sample sizes, and therefore, B% estimation was possible by approximation only.

**Figure 3:**
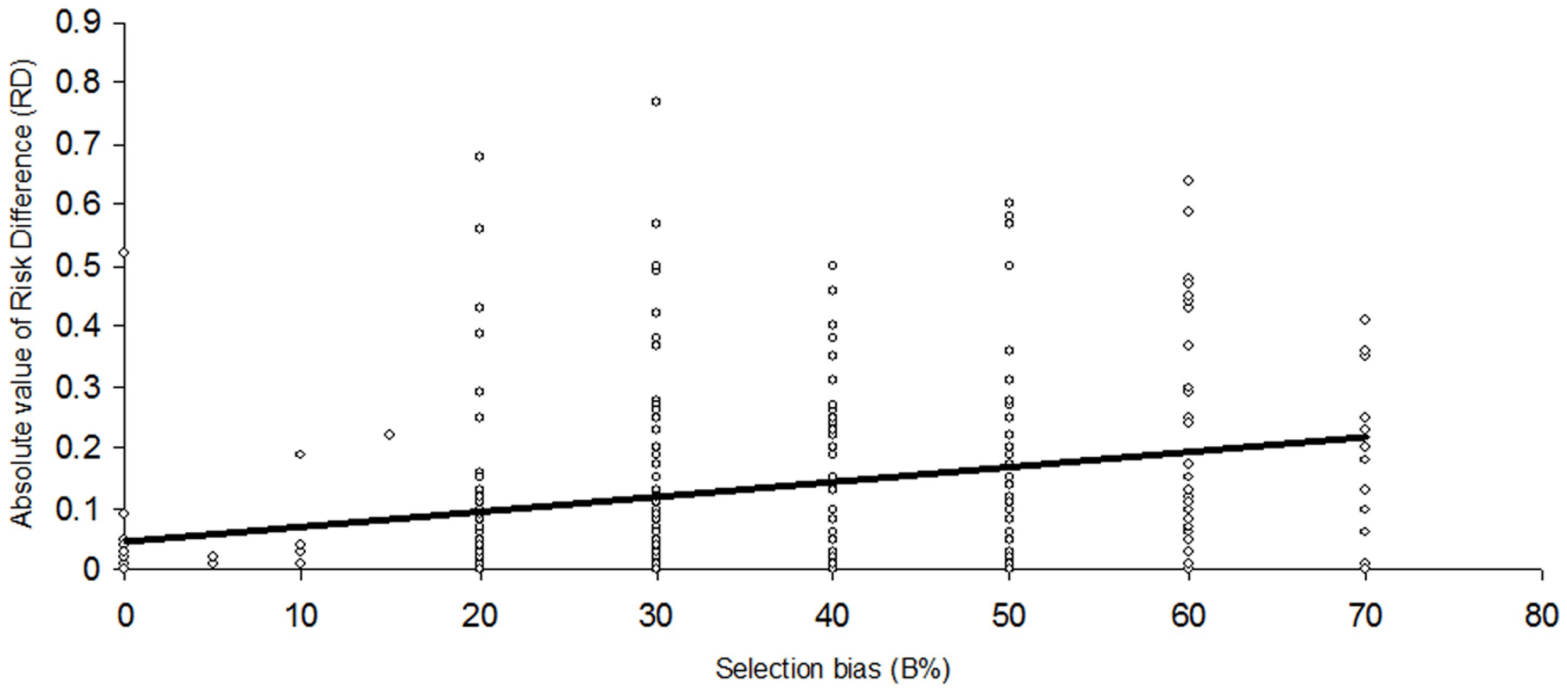
Scatterplot of correlation between trial effect estimates (absolute RD values) and selection bias levels (B%) Image created with MS PowerPoint 2000 SR-1 9.0.3821 (rendered with Adobe Photoshop Elements 11.0)

There was a significant positive correlation between the magnitudes of trial effect estimates (absolute RD values) and the selection bias levels (B% = 0-100) that were identified with the trial-adjusted, SCT-based I^2^ test in 332 RCTs from various medical specialties, published between 1985 and 2023: Spearman’s rho = 0.304, p < 0.0001 (Figure 3). According to Cohen’s guidelines [16], the result indicates a medium-level effect size correlation (0.3 ≤ |r| < 0.5). 

The magnitude of trial effect estimates, represented by the absolute mean RD, of RCTs with identified ‘high’ selection bias (B% 40-100) was 0.18 (SD = 0.16), and that of RCTs with identified ‘low’ selection bias (B% 0-30) was 0.10 (SD = 0.13). The effect estimates were statistically significantly higher (0.094; 95%CI: 0.075 - 0.11; p < 0.0001) for RCTs with identified ‘high’ selection bias than for RCTs with ‘low’ selection bias. The effect size overestimation in RCTs with ‘high’ selection bias, compared to trials with ‘low’ bias, was thus 8.1 (95% CI: 7.5-11.0) percentage points, representing a proportional over-estimation of 86% (95% CI: 80-92%). 

Accordingly, both null hypotheses H01 and H02 were rejected.

Sensitivity analysis results

**Figure 4:**
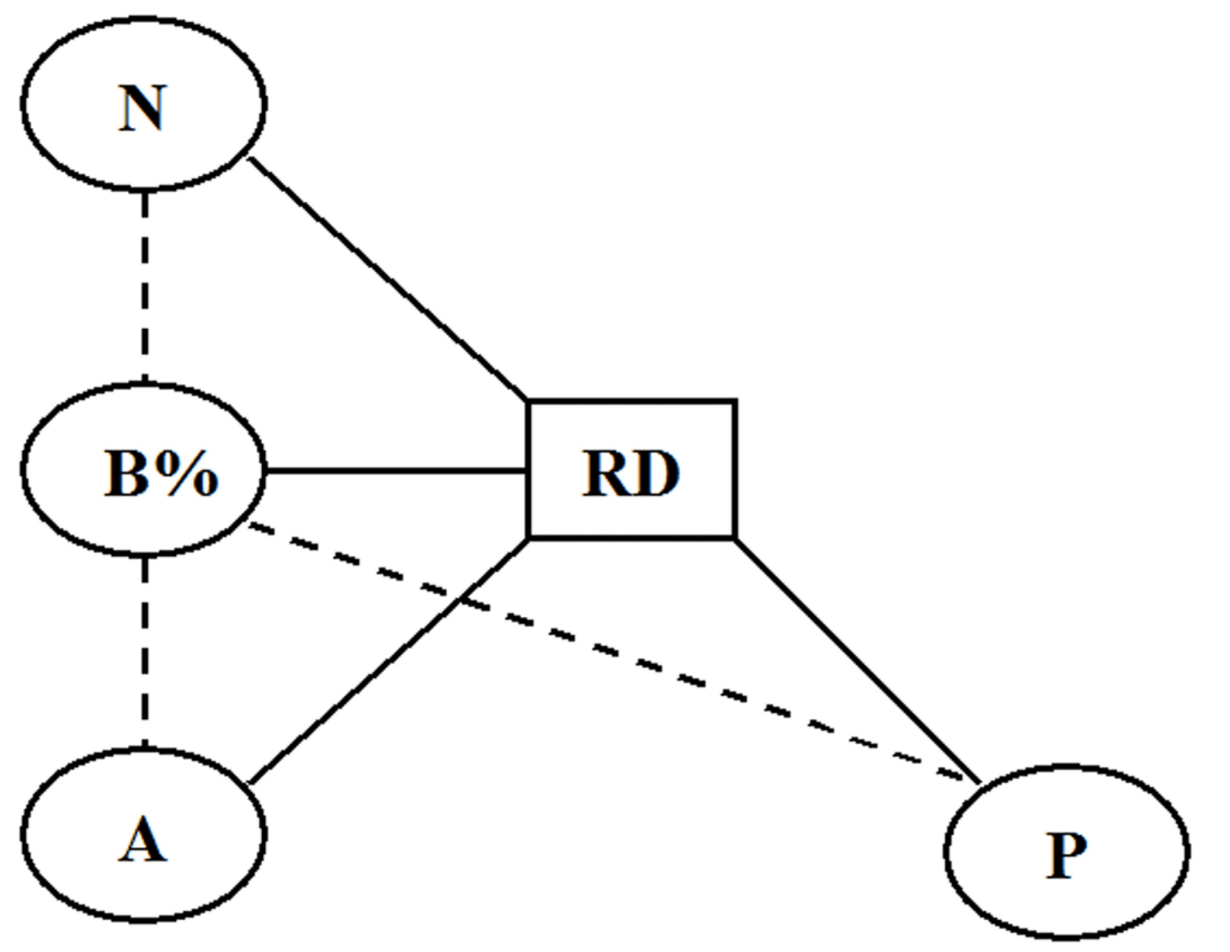
Relationship diagram of potentially confounding factors A: approximate bias estimation, B%: selection bias levels, N: trial sample size, P: comparison against placebo, RD: absolute risk difference Image created with MS PowerPoint 2000 SR-1 9.0.3821 (rendered with Adobe Photoshop Elements 11.0)

Based on a simple relationship diagram (Figure 4), three potential confounding factors were identified that may have directly affected the absolute RD values (RD): RCT sample size per treatment group (N), trial comparison of test interventions against placebo instead of an effective treatment like the current gold standard (P), and B% estimation by approximation outside the pre-specified I^2^ point estimate thresholds (Figure 1) (A).

Sample sizes below and above N = 100 were considered ‘small’ and ‘large’ for 95 and 237 RCTs, respectively. From the 332 tested RCTs, a total of 105 RCTs included comparisons of the test treatment against placebo, and 227 RCTs included comparisons against an active control treatment. Bias testing of 97 RCTs was based on approximate B% estimation, while testing of 235 RCTs followed estimation within pre-specified I^2^ point estimate thresholds (Appendices - Section 5).

The correlation of the factors A, N, and P with either RD or B% and the differences in RD or B% values between RCTs with ‘small’ and ‘large’ sample sizes, placebo and active control treatments, and approximated and I^2^ threshold-guided B% estimation were statistically tested. The test results are presented in Table 1 and Table 2.

**Table 1:** Correlation tests of the confounder effect on the main study results *Spearman correlation A: approximate bias estimation, B%: selection bias level, N: trial sample size per group, P: comparison against placebo

	With RD (absolute)		With B%	
Confounder	r*	p	r*	p
N	0.29	<0.0001**	0.49	<0.0001**
P	0.06	0.28	0.003	0.95
A	0.11	0.046**	0.14	0.01**

**Table 2:** Comparison tests of the confounder effect on the main study results *Statistically significant t: two-tailed t-test, A: approximate bias estimation, B%: selection bias level, CI: confidence interval, MD: mean difference, N: trial sample size per group, P: comparison against placebo, SD: standard deviation, RD: risk difference

Confounder	Comparator 1	Comparator 2	Outcome	t	p	Mean	SD	N	Mean	SD	N	MD	95% CI
N	N>100	N<100	RD	-3.75	0.0002*	0.08	0.13	95	0.15	0.15	237	-0.07	-0.10 to -0.04
	N>100	N<100	B%	-9.76	<0.0001*	22.05	12.71	95	38.99	14.87	237	-16.94	-20.12 to -13.78
P	Yes	No	RD	-1.85	0.06	0.12	0.13	227	0.15	0.18	105	-0.03	-0.07 to 0.01
	Yes	No	B%	-0.04	0.97	34.12	16.51	227	34.19	15.55	105	-0.7	-3.74 to 3.60
A	Yes	No	RD	-2.02	0.044*	0.1	0.14	97	0.14	0.15	235	-0.04	-0.07 to -0.01
	Yes	No	B%	-1.92	0.06	31.5	15.33	97	35.23	16.44	235	-3.73	-7.43 to -0.03*

There was a significant positive correlation between ‘sample size per treatment group’ (N, r = 0.29; p < 0.0001) and a borderline positive correlation between ‘approximate bias estimation’ (A, r = 0.11; p = 0.046), but not between ‘comparisons against placebo’ (P, r = 0.06; p = 0.28) and the absolute RD values. 

An even stronger significant positive correlation between N and selection bias level (B%) was also observed (r = 0.49; p < 0.0001). In addition, statistical comparison of B% values between RCTs with ‘small’ and ‘large’ sample sizes established that the former had significantly larger bias levels than the latter (MD -6.94; 95% CI: -20.12 to -13.78; p < 0.0001).

A significant positive correlation between A and RD (r = 011; p = 0.046), as well as selection bias levels (B%), was also observed (r = 0.14; p < 0.01). Statistical comparison of B% values between RCTs tested, based on approximate B% estimation and B% estimation within pre-specified I^2^ point estimate thresholds, showed that the former resulted in significantly larger bias levels than the latter (MD -3.73; 95% CI: -7.43 to -0.03). This indicates that approximate B% estimation may have yielded erroneously higher B% values, potentially confounding its true association with absolute RD values. Testing the B%/RD relationship in RCTs with B% estimation within pre-specified thresholds still revealed a significant positive correlation (r = 0.25, p < 0.001). However, the difference between effect estimates of RCTs was less for RCTs with identified ‘high’ selection bias in comparison to RCTs with ‘low’ selection bias than that of the main study results (0.07; 95% CI: 0.03-0.11; p = 0.0005). Accordingly, the effect size over-estimation in RCTs with ‘high’ selection bias, compared to trials with ‘low’ bias, was only 7.0 percentage points, representing a proportional over-estimation of 64% only. This suggests that the confounding effect of approximate B% estimation outside the pre-specified I^2^ point estimate thresholds (Figure 1) was 22%.

## Discussion

The objectives of this study were to test two null hypotheses: H01: The magnitude of trial effect estimates is not significantly positively correlated with the selection bias level spectrum, as determined by the trial-adjusted, SCT-based I^2^ test. H02: The magnitude of trial effect estimates does not differ significantly between RCTs with identified ‘low’ and ‘high’ selection biases. Both null hypotheses were rejected.

The observed significantly positively B%/RD correlation (Spearman’s rho = 0.304, p < 0.0001) and the statistically significantly higher absolute RD values (0.0094; 95%CI: 0.075-0.11; p < 0.0001) of RCTs with ‘high’ compared to RCTs with ‘low’ selection bias levels (B%) reconfirmed previous meta-epidemiological study results that high selection bias risk, due to inadequate/unclear (versus adequate) random sequence generation (ratio of odds ratios (ROR) 0.93, 95% CI 0.86 to 0.99) and random allocation concealment (ROR 0.90, 95% CI 0.84 to 0.97), exaggerates intervention effect estimates in clinical trials [17]. Such exaggeration may, depending on the magnitude of the reported effect estimate with its lower and upper confidence levels, change a reported significant result into a non-significant result or even reverse the result’s effect direction.

The results from both null hypothesis tests further suggest that the trial-adjusted, SCT-based I^2^ test in its current version is effective in identifying high-level (B% 40-100) selection bias in RCTs, and such bias may be associated with at least 64% overestimation of the true treatment effect. Based on the results of this study, it is now possible to estimate the extent of selection bias, defined as the percentage of trial subjects from the total RCT sample size non-randomly allocated in favor of one treatment group over the other (B%), and the potential impact of such bias on the reported trial effect estimate, corresponding to a 7.0 percentage point overestimation/proportional overestimation, with less potential confounder effect equating to 64%.

Against this background, the application of the test in future systematic reviews of RCTs may be considered, particularly in view of the observed 27% (RR 0.73; 95%CI: 0.58-0.91; p = 0.005) higher risk for not identifying RCTs with high election bias by using Cochrane’s RoB 2 tool/Domain 1 alone.

However, the results of this study also highlight the limited applicability of the current I^2^ test version. Of 482 RCTs with the patient-level (non-cluster), parallel-study design, reporting clear group allocation and trial outcomes for all randomised patients, only 235 (49%) trials were testable. RCTs could not be tested when they did not report baseline data for each treatment group (n = 89) and when baseline data were not computable (n = 61), i.e., not reported either as a mean value (with SD or SE), or as a median value with the minimum/maximum range or IQR plus sample size per group. Therefore, the quality of RCT reporting has a direct effect on the test’s applicability in practice. The CONSORT statement recommends that a table showing baseline demographic and clinical characteristics for each intervention group should be included when reporting RCTs [18]. Good practice also requires that these characteristics be reported as mean values with SD or SE or median values with minimum/maximum or IQR range. As long as the RCT reporting complies with these recommendations, the current I^2^ test version can be applied.

In addition, during the testing of 97 additional RCTs, the established I^2^ point estimates for all pre-specified sample sizes during meta-analysis did not fall within the specified thresholds, necessitating the authors to 'approximate' the bias levels (B%).

Sensitivity analysis showed that such guessing (Confounder A, Figure 4) was positively correlated with RD (r = 11; p = 0.046) and even more so with B% (r = 14; p < 0.01). Guessing (or approximate estimation) also generated significantly larger B% levels than threshold guided estimation (MD -3.73; 95% CI: -7.43 to -0.03). From these findings, it appears that approximate estimation might have introduced a 22% overestimation into the main study results (RD mean difference between ‘high’ and ‘low’ biased RCTs: 0.094; 95% CI: 0.075-0.11; p < 0.0001 vs. 0.07; 95% CI: 0.03-0.11; p = 0.0005). Therefore, 'approximating' B% values when I^2^ point estimates fall outside pre-specified thresholds (Figure 1) should be avoided. Instead, the test should be repeated using another suitable baseline variable. Baseline variables are suitable if they exhibit an apparently large difference between the compared treatment groups and are considered predictive of the measured trial outcome.

Besides the confounding effect of unguided approximate bias estimation (Confounder A, Figure 4), no such effect was identified through sensitivity analysis for trial sample size and comparison against placebo (Confounders N and P, respectively, Figure 4).

The results of the sensitivity analysis (Tables 1-2) did confirm a positive association of the sample size with trial effect estimates (r = 0.29, p < 0.0001). This is in keeping with the results of a previous systematic review of meta-epidemiological studies [19], reporting the pooled results of two studies including 919 patients, by Zhang et al. (2013) [20] and Dechartres et al. (2013) [21], that indicated a statistically significant larger effect estimates for trials with <100 patients per intervention group with an overestimation of 33% (ROR 0.68; 95% CI: 0.54-0.82; I^2^ = 80.2%) in comparison to trials with at least 100 patients. A further study by Nüesch et al. (2010) also showed a statistically significant higher effect estimate for smaller trials (ES -0.21; 95% CI: -034 to -0.08) [19,22]. However, the sensitivity analysis also revealed a positive association between the sample size (N) and selection bias levels (r = 0.49, p < 0.0001), with statistically higher bias levels (B%) in small trials compared to larger trials: MD -6.94; 95% CI: -20.12 to -13.78; p < 0.0001. These results indicate that RCTs with <100 subjects per group are associated with larger selection bias than larger trials (N > 100), and the increase in B% values strengthened the positive B%/RD correlation. This aligns with the main analysis result for hypothesis H01, suggesting that no confounding effect of N was assumed.

No statistically significant association of trial effect sizes or selection bias levels with placebo comparisons (Confounder P, Figure 4) were identified (Tables 1-2), and therefore, no confounding effect on the main results was assumed in this study.

Study limitations

The results of this study are limited to its underlying database of 332 RCTs, published in various medical specialties over a 38-year period, identified from 141 systematic review reports. These systematic reviews were randomly selected from a larger cohort of 423 reports, which were selected based on a systematic literature search with January 24, 2024, as the cut-off date, making them representative of the prevalence and severity of selection bias in RCTs up to the end of 2023, in general. The underlying database included 2.6 times more RCTs than the minimum sample size calculated for this study, suggesting that the results may be considered to be of high precision. Further research on developing the trial-adjusted, SCT-based I^2^ test for identifying selection bias in single RCTs should revise the pre-specified I^2^ point estimate thresholds (Figure 1) to increase the percentage of trials testable via threshold-guided B% estimation. Further studies may also investigate the relationship between trial effect estimates with each individual bias level.

## Conclusions

Within the limits of this study, the trial-adjusted, SCT-based I^2^ test appeared to be effective in identifying high-level selection bias in RCTs, associated with at least a 64% overestimation of the true treatment effect. Based on this study’s results, it is now possible to estimate the extent of selection bias and the potential impact of such bias on the reported trial effect estimate using the test. However, it has been observed that low RCT reporting quality limits the applicability of the test. Before the test can be recommended for routine use in systematic reviews, further research should revise the pre-specified I^2^ point estimate thresholds to increase the percentage of trials that can be tested based on threshold-guided B% estimation.

## Appendices

All data are made fully available without restriction in the Appendices (Sections 1-5) and can be freely downloaded via the following link: https://data.mendeley.com/datasets/mjfcjr76vx/1.

## References

[1] Higgins JP, Thompson SG (2002). Quantifying heterogeneity in a meta-analysis. Stat Med.

[2] Mickenautsch S, Yengopal V (2024). A test method for identifying selection bias risk in prospective controlled clinical therapy trials using the I2 point estimate. Cureus.

[3] Mickenautsch S, Yengopal V (2024). The I2 test for selection bias risk assessment in single trials: recommended simulated comparator trial (SCT) settings. Cureus.

[4] Mickenautsch S, Yengopal V (2024). Trial-adjusted versus generic simulated comparator trial (SCT) settings for selection bias appraisal using the I2 test. Cureus.

[5] Mickenautsch S, Yengopal V (2025). I2 statistic as a test for selection bias in randomised controlled trials. Cureus.

[6] Mickenautsch S, Yengopal V (2024). Significance testing for differences between baseline variables versus the I2 test in detecting selection bias in randomised controlled trials: a simulation study. Cureus.

[7] Mickenautsch S, Yengopal Y (2025). The I2 statistic as selection bias test: trial effect estimates in relation to identified bias levels -a meta-epidemiological study [PREPRINT]. Authorea.

[8] Moher D, Liberati A, Tetzlaff J, Altman DG (2009). Preferred reporting items for systematic reviews and meta-analyses: the PRISMA statement. PLoS Med.

[9] Mickenautsch S, Yengopal V (2025). The I2 statistic as selection bias test: trial effect estimates in relation to identified bias levels (Protocol). Authorea.

[10] Mickenautsch S, Yengopal V (2024). Selection bias risk in randomized controlled trials rated as low bias using risk of bias, version 2 (RoB2) tool. Cureus.

[11] Sterne JA, Savović J, Page MJ (2019). RoB 2: a revised tool for assessing risk of bias in randomised trials. BMJ.

[12] Hozo SP, Djulbegovic B, Hozo I (2005). Estimating the mean and variance from the median, range, and the size of a sample. BMC Med Res Methodol.

[13] Wan X, Wang W, Liu J, Tong T (2014). Estimating the sample mean and standard deviation from the sample size, median, range and/or interquartile range. BMC Med Res Methodol.

[14] (2008). Review Manager (RevMan) [Computer program]. Version 5.0. Copenhagen: The Nordic Cochrane Centre. https://revman.cochrane.org/info.

[15] Faul F, Erdfelder E, Lang AG, Buchner A (2007). G*Power 3: a flexible statistical power analysis program for the social, behavioral, and biomedical sciences. Behav Res Methods.

[16] Cohen J (1988). Statistical Power Analysis for the Behavioral Sciences.

[17] Page MJ, Higgins JP, Clayton G, Sterne JA, Hróbjartsson A, Savović J (2016). Empirical evidence of study design biases in randomized trials: systematic review of meta-epidemiological studies. PLoS One.

[18] Hopewell S, Chan AW, Collins GS (2025). CONSORT 2025 statement: updated guideline for reporting randomised trials. BMJ.

[19] Mickenautsch S, Rupf S, Miletić I, Yengopal V (2022). Extension of the Composite Quality Score (CQS) as an appraisal tool for prospective, controlled clinical therapy trials - a systematic review of meta-epidemiological evidence. PLoS One.

[20] Zhang Z, Xu X, Ni H (2013). Small studies may overestimate the effect sizes in critical care meta-analyses: a meta-epidemiological study. Crit Care.

[21] Dechartres A, Trinquart L, Boutron I, Ravaud P (2013). Influence of trial sample size on treatment effect estimates: meta-epidemiological study. BMJ.

[22] Nüesch E, Trelle S, Reichenbach S (2010). Small study effects in meta-analyses of osteoarthritis trials: meta-epidemiological study. BMJ.

